# The Murine Factor H-Related Protein FHR-B Promotes Complement Activation

**DOI:** 10.3389/fimmu.2017.01145

**Published:** 2017-09-19

**Authors:** Marcell Cserhalmi, Ádám I. Csincsi, Zoltán Mezei, Anne Kopp, Mario Hebecker, Barbara Uzonyi, Mihály Józsi

**Affiliations:** ^1^MTA-ELTE Lendület Complement Research Group, Department of Immunology, ELTE Eötvös Loránd University, Budapest, Hungary; ^2^Junior Research Group for Cellular Immunobiology, Leibniz Institute for Natural Product Research and Infection Biology – Hans Knöll Institute, Jena, Germany; ^3^MTA-ELTE Immunology Research Group, Department of Immunology, ELTE Eötvös Loránd University, Budapest, Hungary

**Keywords:** complement deregulation, C-reactive protein, factor H, factor H-related protein, endothelial cell, extracellular matrix, necrotic cell, pentraxin 3

## Abstract

Factor H-related (FHR) proteins consist of varying number of complement control protein domains that display various degrees of sequence identity to respective domains of the alternative pathway complement inhibitor factor H (FH). While such FHR proteins are described in several species, only human FHRs were functionally investigated. Their biological role is still poorly understood and in part controversial. Recent studies on some of the human FHRs strongly suggest a role for FHRs in enhancing complement activation *via* competing with FH for binding to certain ligands and surfaces. The aim of the current study was the functional characterization of a murine FHR, FHR-B. To this end, FHR-B was expressed in recombinant form. Recombinant FHR-B bound to human C3b and was able to compete with human FH for C3b binding. FHR-B supported the assembly of functionally active C3bBb alternative pathway C3 convertase *via* its interaction with C3b. This activity was confirmed by demonstrating C3 activation in murine serum. In addition, FHR-B bound to murine pentraxin 3 (PTX3), and this interaction resulted in murine C3 fragment deposition due to enhanced complement activation in mouse serum. FHR-B also induced C3 deposition on C-reactive protein, the extracellular matrix (ECM) extract Matrigel, and endothelial cell-derived ECM when exposed to mouse serum. Moreover, mouse C3 deposition was strongly enhanced on necrotic Jurkat T cells and the mouse B cell line A20 by FHR-B. FHR-B also induced lysis of sheep erythrocytes when incubated in mouse serum with FHR-B added in excess. Altogether, these data demonstrate that, similar to human FHR-1 and FHR-5, mouse FHR-B modulates complement activity by promoting complement activation *via* interaction with C3b and *via* competition with murine FH.

## Introduction

The proper balance between enhancement and inhibition of complement activation is important to maintain the physiological functions of complement and prevent pathological complement activation and complement-mediated diseases (1). Among the complement regulatory proteins that protect host tissues, factor H (FH) is the main soluble inhibitor of the alternative complement pathway and the amplification loop. By hindering the assembly and accelerating the decay of the C3bBb alternative pathway C3 convertase enzyme and by acting as a cofactor for the factor I-mediated cleavage of C3b, FH prevents overactivation of the system (2, 3).

Factor H-related (FHR) proteins have been described in several species, including the fish barred sand bass, zebrafish, mice, rats, and humans (4–7), but these complement proteins were scarcely studied (8). The number of *CFHR* genes differs among these species and direct homologs of the human FHRs cannot be identified in lower vertebrates (8, 9). Various isoforms of the FHRs also exist that require further characterization in terms of functional significance (9–12). To date, the five human FHRs are best characterized; still, their biological function is poorly understood [reviewed in Ref. (8, 13, 14)]. Most, particularly early, studies assessed the direct complement regulatory roles of FHRs, and some activities in the regulation of C3 or C5 convertases (15–18), inhibition of the terminal pathway by FHR-1 (19), and synergistic enhancement of the cofactor activity of FH by FHR-3 and FHR-4 (15) were reported. Recent studies, however, highlight a paradigm change, and described deregulation, i.e., competitive inhibition of FH, as a major function of FHR-1, FHR-2, and FHR-5 (20–24). FHR-3 was described to compete off FH from binding to fHbp of *Neisseria meningitidis* (25). In addition, FHR-1, FHR-4, and FHR-5 were shown to promote alternative pathway activation by binding C3b and allowing formation of the C3bBb alternative pathway C3 convertase enzyme (23, 24, 26), FHR-5 also *via* interaction with properdin (27). In addition, FHR-1 was shown to modulate activation of human neutrophils in the context of interaction of neutrophils with the human-pathogenic yeast *Candida albicans* (28), and, by binding C3d, FHR-3 to inhibit C3d-mediated co-activation of B cells (29).

In FH, the N-terminal domains mediate the complement inhibitor functions of the protein, and the CCP7 as well as the C-terminal domains CCPs 18–20 mediate interactions with ligands, such as pentraxins, heparin, and the host cell markers sialic acid/glycosaminoglycans (2, 3, 30, 31). FH also interacts with C3b *via* multiple sites, located in CCPs 1–4 and 19–20 (32). The dual recognition of polyanionic host cell markers and deposited C3b/C3d on host cells under complement attack allows FH for potent complement inhibition on such host surfaces (33, 34). The homology between FHRs and FH suggests similar or overlapping ligand binding capacities and functions; however, the FHRs do lack the N-terminal complement inhibitor domains of FH. Interaction with C3b, heparin and the pentraxins C-reactive protein (CRP) and pentraxin 3 (PTX3) and binding to necrotic cells were described for one or more of the human FHR proteins (12, 15–17, 19, 23, 24, 27, 35, 36).

While understanding the exact biological roles of the FHRs requires further investigation, human genetic disease-association studies strongly implicate a role of the FHR proteins in the modulation of complement activation [reviewed in Ref. (13, 37)]. Characterization of disease-associated FHR variants indicated that they likely cause enhanced alternative complement pathway activation (20–22, 27). Recently, the lack of murine FHR-C was linked to susceptibility to autoimmunity (38).

**Figure 1:**
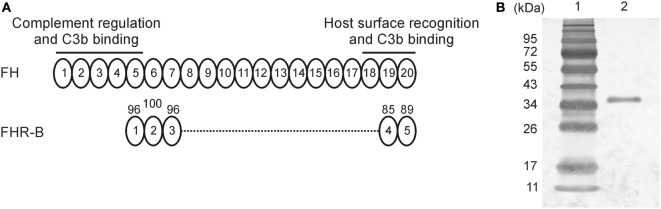
Expression and purification of factor H-related (FHR)-B. **(A)** Schematic drawing of murine factor H (FH) and FHR-B. Factor H is built up of 20 CCP domains, of which CCPs 1–5 mediate complement regulatory activity and CCPs 18–20 mediate surface recognition; both functional regions interact with mouse and human C3b (39). The FHR-B domains are shown aligned with the corresponding homologous domains of factor H. The numbers above the domains indicate the percentage of amino acid sequence identity. **(B)** Murine FHR-B was expressed in insect cells and purified by nickel-affinity chromatography. The purity was assessed by silver staining. 1 µg purified recombinant FHR-B (lane 2) was run on 10% SDS-PAGE and stained with silver nitrate. The molecular weight marker (lane 1) is indicated on the left.

In mice, various FHR transcripts have been reported (6), but only FHR-B and FHR-C were studied at the protein level (9). These murine FHRs have been shown to bind to human C3b, heparin and human umbilical vein endothelial cells (HUVECs) from mouse serum. The FHR-B protein is composed of five CCP domains that are homologous to FH CCPs 5, 6, 7, 19, and 20, with 96, 100, 96, 85, and 89% amino acid sequence identity, respectively (6, 9) (Figure 1A). Thus, this murine FHR protein—similar to its human counterparts—lacks domains homologous to the C3b binding and complement regulatory N-terminal domains of FH, but include the FH-homolog domains that were identified to be responsible for interactions with human and mouse C3b, heparin and endothelial cells (i.e., CCPs 18–20) (39). FHR-B was previously shown to be present in the plasma of various mouse strains (9). A recombinant form of FHR-B was expressed in the yeast *Pichia pastoris*, purified by heparin affinity chromatography, and showed in ELISA to bind human C3b. However, the protein was highly glycosylated and required enzymatic removal of the carbohydrate chains (9).

Mice may represent a model organism to investigate the physiological and pathological roles of the FHR proteins *in vivo*. Therefore, the aim of this study was to assess whether FHR-B shares functions recently attributed to human FHR proteins, such as interactions with pentraxins, the extracellular matrix (ECM), and necrotic cells, and facilitation of complement activation.

## Materials and Methods

### Proteins, Antibodies, and Sera

Recombinant mouse FHR-B was generated using the pBSV-8His Baculovirus expression vector (40), expressed in *Spodoptera frugiperda* (Sf9) cells, and purified by nickel-affinity chromatography (40). Recombinant murine FH15–20, PTX3, CRP, and biotinylated goat anti-mouse PTX3 antibody were obtained from R&D Systems (Biomedica, Budapest, Hungary). The monoclonal rat anti-mouse FH antibody 5C2 (generated against mouse FH1–5) was previously described (39).

Purified human FH, C3, C3b, factor B (FB), factor D (FD), properdin [factor P (FP)], C1q, and goat anti-human FB antiserum were obtained from Merck Ltd. (Budapest, Hungary). The C3a EIA kit and the anti-human FH mAb A254 were from Quidel (Biomedica, Budapest, Hungary). Matrigel was from Sigma-Aldrich Ltd. (Budapest, Hungary). Horseradish peroxidase (HRP)-conjugated goat anti-human C3 was from MP Biomedicals (Solon, OH, USA). HRP-conjugated swine anti-rabbit immunoglobulins, rabbit anti-goat immunoglobulins and goat anti-mouse immunoglobulins were from Dako (Hamburg, Germany). The HRP- and FITC-conjugated anti-mouse C3 antibodies were kind gifts of Drs. Anna Erdei and József Prechl (Eötvös Loránd University, Budapest), respectively. Mouse serum was from PAA Laboratories (Pasching, Austria).

### Microtiter Plate Binding Assays

Interaction of FHR-B with C3b was measured in Dulbecco’s PBS containing Ca^2+^ and Mg^2+^ [Dulbecco’s phosphate-buffered saline (DPBS)^++^; Lonza, Cologne, Germany]. FHR-B, mouse FH15–20 and human serum albumin (HSA) as control protein were immobilized at 5 µg/mL in microplate wells and, after blocking with 3% BSA in DPBS^++^, incubated with up to 10 µg/mL human C3b for 1 h at 22°C. C3b binding was detected with HRP-conjugated goat anti-human C3 antibody. To measure competition between FHR-B and human FH, human C3b was immobilized in microplate wells at 5 µg/mL. After blocking, FHR-B in 40 µg/mL final concentration and human FH (in 20 µg/mL) were added for 45 min at 22°C. FH binding was detected using the FH-specific monoclonal antibody A254.

To measure PTX3 binding, FHR-B, C1q (as positive control), and bovine serum albumin (BSA; as negative control) were immobilized at 5 µg/mL. After washing and blocking with 3% BSA, 5 µg/mL recombinant murine PTX3 was added for 1 h at 20°C, and PTX3 binding was detected using biotinylated antimouse PTX3 antibody and HRP-conjugated streptavidine.

### C3 Convertase Assays

Formation of human C3bBb alternative pathway C3 convertase on surface-bound mFHR-B and detection of the C3 convertase assembly using anti-FB polyclonal antibody were performed as previously described (26). Briefly, microtiter plate wells were coated with 5 µg/mL FHR-B, FHR-4B, BSA, and C3b. After blocking with 4% BSA, 10 µg/mL human C3b was added for 1 hr at 22°C, then the wells were incubated with purified human factors B, D, and P in convertase buffer (4% BSA, 0.05% Tween-20, and 2 mM Ni^2+^) for 30 min at 37°C. The formed C3bBb was detected using anti-FB antiserum (1,000×) and a corresponding secondary Ab (1,000×). The convertase activity was measured by adding 10 µg/mL purified human C3 for 1 hr at 37°C and quantifying the generated C3a by a C3a ELISA kit (Quidel).

### Complement Activation Assays

Nunc microtiter plate wells were coated with 5 µg/mL FHR-B and BSA in DPBS, and, after blocking with 5% BSA in DPBS containing 0.05% Tween-20, incubated with 10% mouse serum with or without 5 mM Mg^2+^-EGTA or 20 mM EDTA for 30 min at 37°C. Deposition of mouse C3-fragments was detected using HRP-conjugated mouse C3-specific antibody.

In other experiments, Nunc microplate wells were coated with 5 µg/mL mouse PTX3, mouse CRP and the ECM extract Matrigel diluted 1:30 in DPBS. After blocking with 5% BSA in DPBS containing 0.05% Tween-20, 10% mouse serum was added in 5 mM Mg^2+^-EGTA or 20 mM EDTA with or without 10 µg/mL FHR-B and HSA for 30 min at 37°C. Complement activation was detected by measuring deposition of C3 fragments using HRP-conjugated anti-mouse C3 antibody.

Endothelial cell-derived ECM was prepared by culturing HUVEC (Lonza) according to the manufacturer’s instructions in EBM-2 medium (Lonza) on gelatin-coated 96-well tissue culture plates (0.2% gelatin) in a cell incubator with humidified atmosphere containing 5% CO_2_ for 7 days at 37°C. Cells were washed and detached from the plate by incubation in DPBS containing 20 mM EDTA at 37°C. The cell-free ECM was blocked with 4% BSA in 0.05% DBPS-Tween. 5% mouse serum was added in 5 mM Mg^2+^-EGTA, DBPS^+ +^ or DPBS containing 20 mM EDTA for 30 min at 37°C with or without 5 and 10 µg/mL FHR-B and 10 µg/mL HSA as control. Complement activation was detected by measuring deposition of C3 fragments using HRP-conjugated mouse C3-specific goat antibody.

### Binding of FHR-B to Necrotic Cells and Measurement of C3 Deposition from Serum on Necrotic Cells

To investigate FHR-B binding and complement activation on necrotic cells, necrosis of Jurkat T cells and A20 murine B cells was induced by heating at 65°C for 30 min. Necrotic Jurkat T cells were incubated with 20 µg/mL FHR-B for 30 min at 37°C. Binding was measured by flow cytometry using rat anti-mouse FH antibody (5C2) and Alexa647-conjugated goat anti-rat IgG (Invitrogen, Thermo Fisher Scientific, Waltham, MA, USA). A total of 10,000 cells was measured using a FACSCalibur flow cytometer (BD Biosciences, Heidelberg, Germany) and data were analyzed using FlowJo software (TreeStar, Ashland, OR, USA). To measure complement activation, necrotic cells were incubated with 20% mouse serum with or without 20 µg/mL FHR-B in RPMI-1640 medium containing 10% FCS. After 30 min at 37°C, the cells were washed with DPBS and labeled with FITC-conjugated anti-mouse C3. Cells were gated based on morphology and staining with propidium iodide. Data were collected and analyzed using a FACSCalibur instrument and the FlowJo Software.

### Hemolysis Assay and FHR-B Binding to Sheep Red Blood Cells (SRBCs)

To determine whether FHR-B causes anomalous lysis, SRBCs (Culex Bt., Budapest, Hungary) were washed three times in veronal buffer containing 10 mM Mg^2+^-EGTA (Lonza). FHR-B and mouse FH15–20 were added to 2% SRBCs and 20% mouse serum in a final volume of 60 µL in veronal buffer containing 10 mM Mg^2+^-EGTA, and incubated at 37°C for 30 min with gentle shaking (400 rpm). The red cells were sedimented by centrifugation and the released hemoglobin was measured at 405 nm.

After the SRBC samples were washed and lysed, the lysates were subjected to 10% SDS-PAGE and Western blotting and FHR-B binding was detected using the 5C2 antibody and HRP-conjugated goat anti-rat IgG.

### Statistical Analysis

Statistical analysis was performed using GraphPad Prism version 4.00 for Windows (GraphPad Software, San Diego, CA, USA). A *p* value < 0.05 was considered statistically significant.

## Results

### Generation and Recombinant Expression of FHR-B in Insect Cells

In our previous study, FHR-B was expressed in yeast that resulted in overglycosylation of the protein and the need to remove glycan chains (9). For the current study, FHR-B was cloned into the Baculovirus expression vector pBSV-8His (40). The translated recombinant protein has a theoretical pI of 7.8 and a predicted molecular mass of 35,720 Da (38,378 Da with the His-tag). The protein was expressed in Sf9 insect cells and purified by nickel-affinity chromatography (Figure 1B).

### FHR-B Binds to Human C3b and Competes with FH

**Figure 2:**
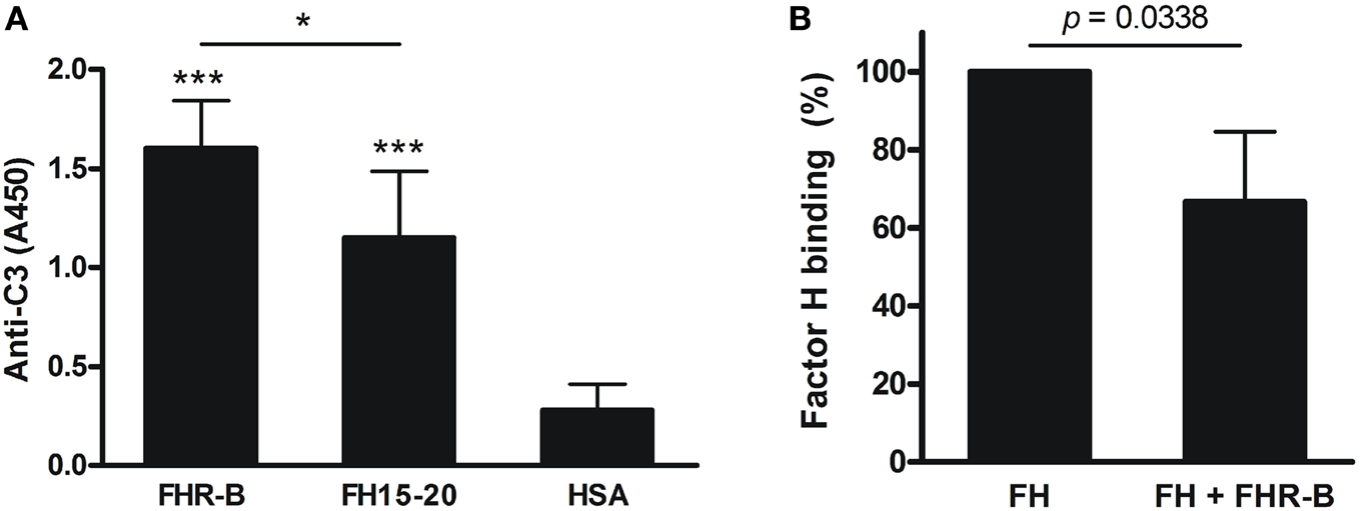
Interaction of factor H-related (FHR)-B with C3b and competition with human factor H. **(A)** Binding of human C3b to recombinant FHR-B and to mouse FH15–20 was measured in ELISA. 5 µg/mL FHR-B, mouse FH15–20 and human serum albumin (HSA) as control protein were immobilized in microplate wells, then 10 µg/mL purified human C3b was added for 1 h at 22°C. C3b binding was detected using horseradish peroxidase (HRP)-conjugated goat anti human C3 antibody. Data are mean absorbance values + SD derived from five independent experiments. *p < 0.05, ***p < 0.001, one-way ANOVA. **(B)** The binding of 20 µg/mL human factor H to human C3b was measured in the presence of FHR-B at 40 µg/mL final concentration, using a factor H (FH)-specific mAb (A254) for detection. Data are mean absorbance values + SD derived from four independent experiments. *p < 0.05, paired *t*-test.

Factor H-related-B from serum and FHR-B expressed in yeast were shown to bind weakly to human C3b (9). Mouse FH and its carboxyl-terminal construct (CCPs 18–20) were shown to bind to both mouse and human C3b (39). Based on these previous findings and the conservation of the C-terminal FH domains in FHR-B, and because of the lack of highly purified and well-characterized mouse C3b, we first investigated the interaction of FHR-B expressed in insect cells with human C3b. To this end, FHR-B and the recombinant murine FH C-terminal fragment FH15–20 were immobilized in equimolar amounts in microplate wells, and binding of human C3b was measured. Both murine proteins bound human C3b, thus we could confirm the binding of recombinant FHR-B to C3b in ELISA (Figure 2A). Interestingly, FHR-B bound C3b stronger under these experimental conditions than mouse FH15–20.

Because some of the human FHR proteins were shown to compete with FH for C3b binding, we performed a competition experiment to determine whether FHR-B was also capable of such competition. Human C3b was immobilized in microplate wells, and binding of human FH in the absence and presence of FHR-B was measured with a FH-specific monoclonal antibody. As expected from the C3b binding capacity of FHR-B, in its presence FH binding to C3b was reduced (Figure 2B).

### FHR-B Supports Formation of the C3bBb Convertase *via* C3b Binding, and Promotes Complement Activation

**Figure 3:**
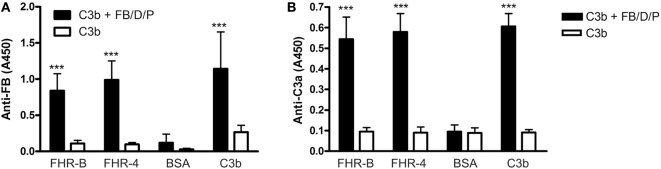
Assembly of the alternative pathway C3 convertase on factor H-related (FHR)-B. **(A)** Assembly of the C3bBb convertase on FHR-B. 5 µg/mL recombinant FHR-B, human FHR-4B (FHR-4), C3b as positive control and BSA as negative control were immobilized in microplate wells. After blocking, the wells were incubated with 10 µg/mL C3b for 1 h at 37°C. The alternative pathway C3 convertase was built up by adding purified factor B (FB), factor D (FD), and factor P (FP) for 30 min. The convertase was detected using a polyclonal anti-FB antiserum. Data are mean absorbance values + SD derived from four independent experiments. ***p < 0.001, one-way ANOVA. **(B)** Activity of the FHR-B bound convertase was measured by adding 10 µg/mL C3 to the wells for 1 h at 37°C. C3a generation was measured by Quidel’s C3a ELISA kit. Data are mean absorbance values + SD derived from four independent experiments. ***p < 0.001, one-way ANOVA.

FHRs lack the C3b and C3 convertase regulating activities of FH but it was demonstrated for FHR-1, FHR-4, and FHR-5 that C3b binding to these FHRs can allow the formation of a fully active C3bBb convertase (23, 24, 26). We therefore tested whether FHR-B was also able to support convertase formation. To this end, FHR-B was immobilized in microplate wells and formation of C3bBb *in vitro* was measured by sequential incubation with human C3b, FB, FD, and properdin. Significant amount of C3bBb was formed on FHR-B in this assay, similar to that of the control protein FHR-4B (Figure 3A). The convertase formed on FHR-B was functionally active as demonstrated by the generation of C3a from human C3 (Figure 3B), suggesting that similar to the human FHR-4 and FHR-5 proteins, mouse FHR-B is able to support activation of the alternative pathway.

**Figure 4:**
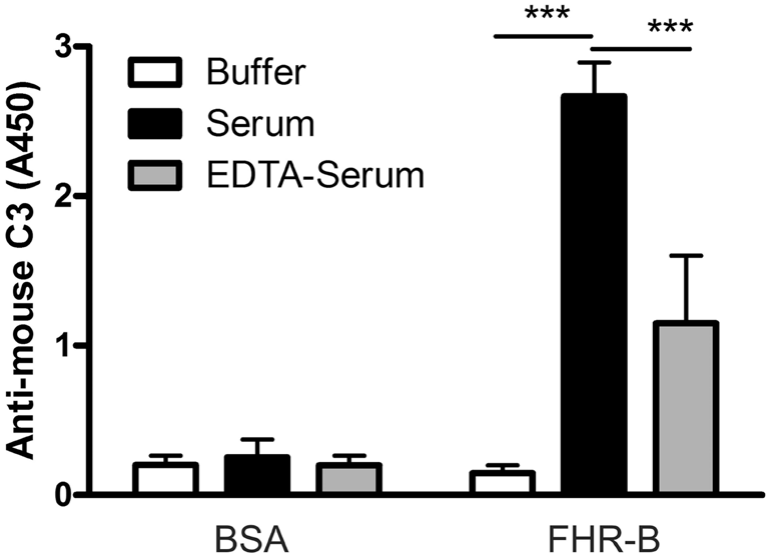
Complement activation by factor H-related (FHR)-B. FHR-B was immobilized on microplate wells and incubated with 10% mouse serum in 5 mM Mg^2+^-EGTA buffer to allow activation of only the alternative pathway or with 10% mouse serum in 20 mM EDTA buffer to inhibit complement activation. C3 deposition was detected using horseradish peroxidase (HRP)-conjugated mouse C3-specific antibody. Data are mean absorbance values + SD derived from three independent experiments. ***p < 0.001, one-way ANOVA.

Therefore, we also tested whether complement activation on FHR-B occurs in serum. Wells were coated with FHR-B, and with BSA as negative control, and incubated with mouse serum containing Mg^2+^-EGTA to allow activation of only the alternative pathway or containing 20 mM EDTA to block complement activation. The deposition of mouse C3 fragments was detected by ELISA. There was a strong and significant C3 deposition on immobilized FHR-B when incubated in serum. The C3 signal in the EDTA-serum sample, i.e., in the absence of complement activation, which likely represents C3 fragments that bound from serum, was significantly reduced (Figure 4).

### FHR-B Binds to Pentraxin 3

**Figure 5:**
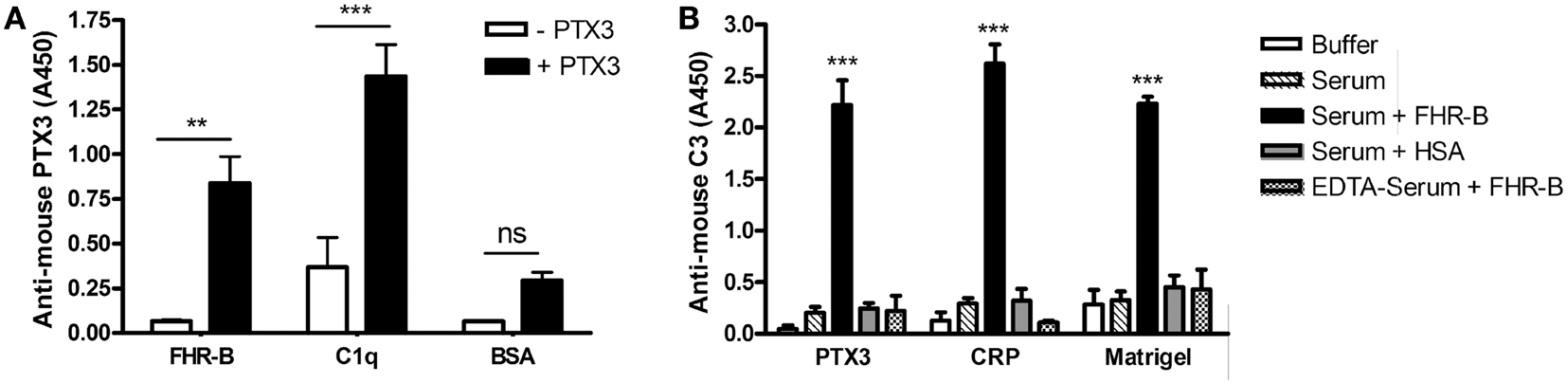
Factor H-related (FHR)-B binds to mouse PTX3 and causes enhanced C3 fragment deposition on pentraxins and Matrigel. **(A)** Microplate wells were coated with FHR-B, human C1q and BSA. After blocking, 5 µg/mL mouse PTX3 was added, and PTX3-binding was detected with a biotinylated anti-mouse PTX3 antibody followed by horseradish peroxidase (HRP)-conjugated streptavidine. Data are mean absorbance values + SEM derived from three independent experiments. **p < 0.01 and ***p < 0.001, one-way ANOVA. **(B)** Nunc microplate wells were coated with 5 µg/mL mouse PTX3, 5 µg/mL mouse C-reactive protein (CRP) and Matrigel diluted 1:30 in Dulbecco’s phosphate-buffered saline (DPBS). After blocking, 10% mouse serum was added in 5 mM Mg^2+^-EGTA buffer or in 20 mM EDTA buffer for 30 min at 37°C with or without 10 µg/mL FHR-B or human serum albumin (HSA), as indicated. Complement activation was detected using HRP-conjugated mouse C3-specific antibody. Data are mean absorbance values + SD derived from three independent experiments. ***p < 0.001, one-way ANOVA.

Because the C-terminal domains of human FHR-1 and FH, as well as domain 7 of FH were shown to include a PTX3 binding site (12, 24, 41), we investigated in ELISA whether murine PTX3 could bind to FHR-B. To this end, FHR-B was immobilized in microplate wells and, after blocking, incubated with recombinant mouse PTX3. We found that this conserved pentraxin interacts with FHR-B, as well as with human C1q, used as a positive control, but it did not bind to BSA, which was used as a negative control (Figure 5A).

### FHR-B Enhances Complement Activation on Pentraxins and ECM

Recent evidence support a role for some of the FHR proteins as competitive inhibitors of FH on certain ligands, such as C3b, pentraxins and the ECM (20, 23, 24). Therefore, we investigated whether FHR-B had the capacity to enhance complement activation on PTX3. To this end, murine PTX3 was immobilized in microplate wells, and incubated in mouse serum. When recombinant FHR-B was added, increased amount of deposited mouse C3 fragments was detected compared with control when HSA was added to the mouse serum (Figure 5B). FHR-B similarly caused enhanced C3 fragment deposition in wells coated with murine CRP and the ECM extract Matrigel (Figure 5B). In mouse serum containing EDTA to block complement activation the addition of FHR-B did not enhance C3 deposition.

**Figure 6:**
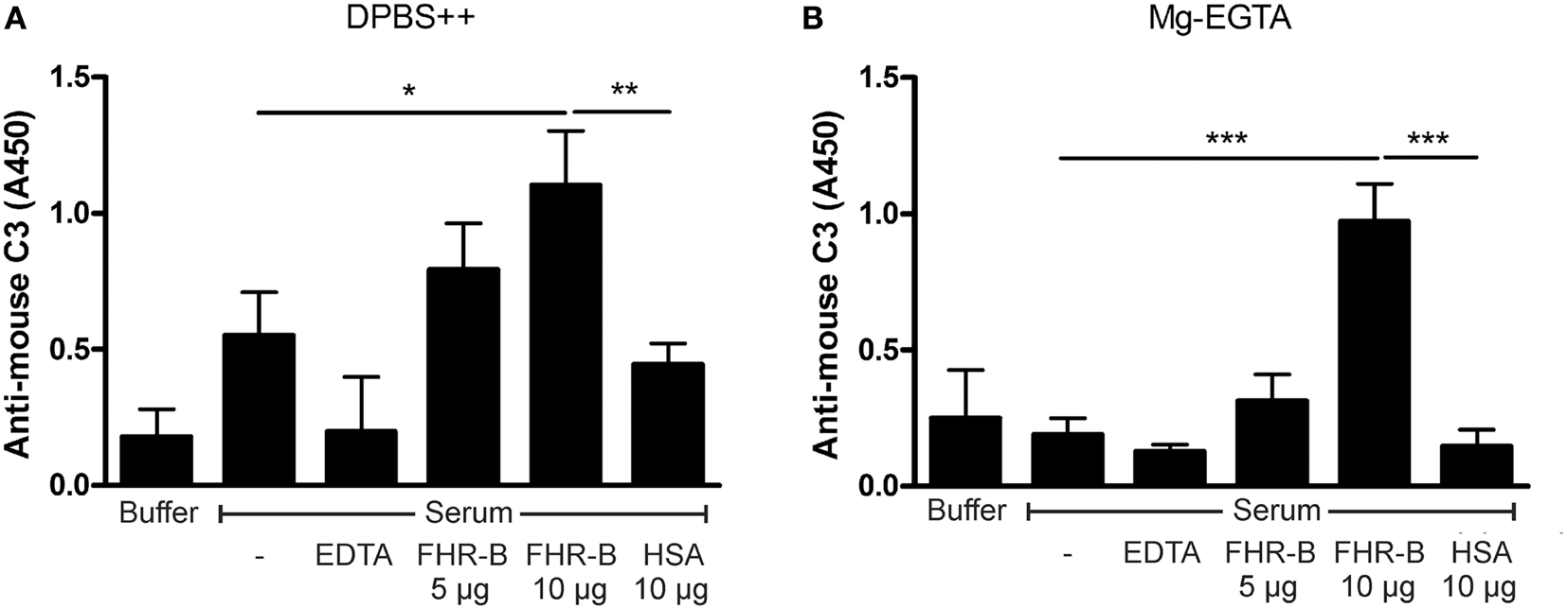
Factor H-related (FHR)-B enhances complement activation on human umbilical vein endothelial cell (HUVEC)-derived extracellular matrix (ECM). HUVEC cells were cultured on gelatin-coated 96-well tissue culture plates for 7 days. After removal of the cells, the cell-derived ECM was blocked, washed and incubated with 5% mouse serum in Dulbecco’s phosphate-buffered saline (DPBS)^++^ **(A)** or in DPBS containing 5 mM Mg^2+^-EGTA **(B)** for 30 min at 37°C with 5 and 10 µg/mL FHR-B or 10 µg/mL human serum albumin (HSA) as control. As an additional negative control, serum diluted in 20 mM EDTA buffer was used to block complement activation. Complement activation was detected by measuring C3-deposition using horseradish peroxidase (HRP)-conjugated mouse C3-specific antibody. Data are mean absorbance values + SD derived from three independent experiments. *p < 0.05, ***p < 0.001, one-way ANOVA.

Complement activation was also investigated on ECM produced *in vitro* by HUVECs, as previously described (12). When the washed HUVEC-ECM was incubated in 10% mouse serum diluted in DPBS^++^, slight C3 deposition was observed, which was significantly increased when recombinant FHR-B, but not when HSA, was added (Figure 6A). Similarly, when the HUVEC-ECM was incubated in 10% mouse serum diluted in Mg/EGTA buffer to allow only alternative pathway activation, FHR-B but not HSA caused enhanced C3 deposition, indicating alternative pathway activation on this matrix (Figure 6B).

### FHR-B Enhances C3-Fragment Deposition on Necrotic Cells

**Figure 7:**
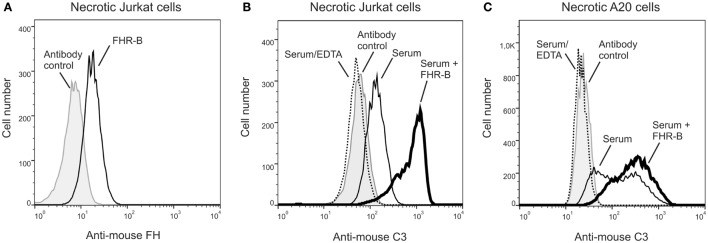
Factor H-related (FHR)-B binds to necrotic cells and increases C3-fragment deposition. **(A)** Binding of 20 µg/mL (~0.5 μM) recombinant FHR-B to necrotic Jurkat T cells was measured by flow cytometry using the 5C2 monoclonal anti-mouse factor H antibody. Representative results of two experiments are shown. Necrotic Jurkat T cells **(B)** and necrotic A20 murine B cells **(C)** were incubated with 20% mouse serum with or without 20 µg/mL FHR-B for 30 min at 37°C. After washing, complement activation was detected by flow cytometry using FITC-conjugated mouse-C3 specific antibody. Representative results of two experiments are shown.

We also investigated whether FHR-B enhances opsonization of dead cells. First, the binding of recombinant FHR-B to Jurkat T cells, in which necrosis was induced by heat treatment, was measured by flow cytometry. FHR-B showed specific binding to necrotic Jurkat cells (Figure 7A).

To measure opsonization on dead cells, necrosis was induced by heat treatment in Jurkat T cells and A20 murine B cells, then the cells were exposed to 20% mouse serum and C3-fragment deposition was monitored by flow cytometry. In both cases, serum treatment resulted in increased C3 deposition compared with the control serum containing EDTA, and this was further enhanced by the addition of recombinant FHR-B (Figures 7B,C). Similar results were obtained in experiments where the necrotic cells were first treated with recombinant FHR-B and then, after washing, were exposed to mouse serum as above (data not shown).

### FHR-B Binds to and Enhances Lysis of Host-Like Cells

**Figure 8:**
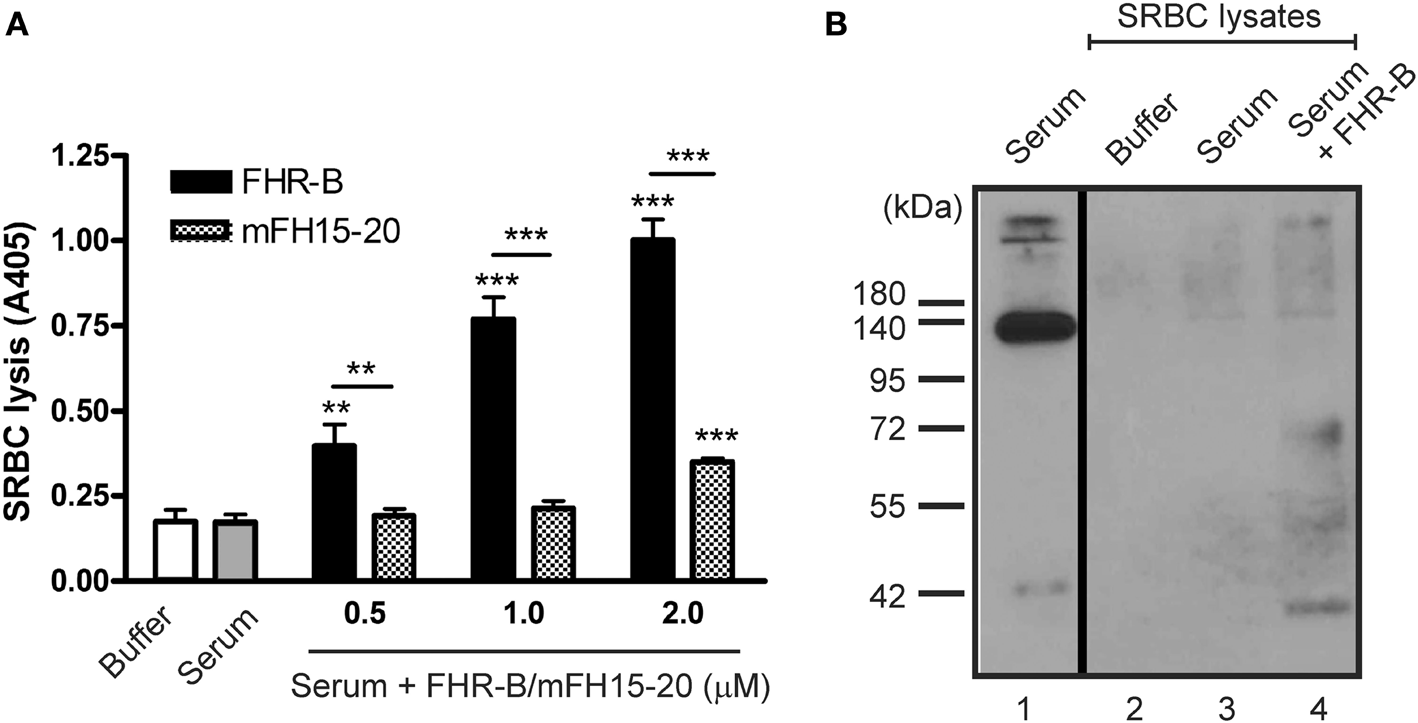
Factor H-related (FHR)-B induces complement-mediated lysis of sheep red blood cells (SRBCs). **(A)** 2% SRBC were incubated in 10 mM Mg^2+^-EGTA containing buffer, 20% mouse serum alone or with increasing concentrations of FHR-B or mouse FH15–20 at 37°C for 30 min. The released hemoglobin in the supernatants was measured at 405 nm. Data are mean absorbance values + SD of three independent experiments (**p < 0.01 and ***p < 0.001, two-way ANOVA). **(B)** Binding of FHR-B to SRBCs. SRBCs were incubated in 20% mouse serum with or without 0.5 µM FHR-B. SRBCs were then washed and lysed and the lysates were subjected to 10% SDS-PAGE and Western-blotting using the 5C2 monoclonal anti-mouse factor H antibody.

Next, we assessed whether FHR-B *via* modulation of complement activation influences the complement-mediated lysis of host cells. To this end, sheep erythrocytes were incubated in mouse serum in the absence or presence of recombinant FHR-B. FHR-B induced dose-dependent lysis of SRBCs measured by the release of hemoglobin (Figure 8A). The FH15–20 fragment, used as a control, had only minor effect under these conditions. Binding of recombinant FHR-B was detected on the lysed SRBCs by Western blotting (Figure 8B).

## Discussion

The possible biological roles of human FHR proteins, due to their disease associations, have gained increasing attention in recent years (13, 14). In contrast to the human ones, murine FHRs were functionally not yet studied. However, in order to properly interpret animal disease models, a better understanding of the functions of non-human FHRs is required, to reveal similarities and differences in their functions in comparison with their human counterparts.

Although there are some controversies regarding the roles of human FHR proteins, a main function identified by recent studies is that they compete with FH for certain self and non-self ligands, such as C3b deposited on surfaces, pentraxins, ECM and, in the case of FHR-3, the FH-binding protein of *N. meningitidis* (20–25). In addition to this indirect role in the enhancement of complement activation, some FHRs may directly activate complement by serving as a platform for alternative pathway convertase formation and are able to promote complement activation on host ligands and surfaces (23, 24, 26).

In this study, we functionally characterized the murine FHR protein, FHR-B. We identified host ligands of FHR-B, such as murine pentraxins, ECM, and necrotic cells, which are likely relevant in the context of mouse models of human diseases. Previously, binding of FHR-B to human C3b was described (9). In the current study, we showed that, similar to the human FHR-1 and FHR-5, this murine FHR protein is able to compete with FH for C3b binding. Interestingly, there was significantly stronger binding of C3b to FHR-B than to the FH15–20 in our assay (Figure 2), which likely explains its strong effect in inducing lysis of the host-like cells SRBC in mouse serum (Figure 8). Furthermore, FHR-B, similar to the human FHR-1, FHR-4, and FHR-5 proteins, supports the assembly of the alternative pathway C3 convertase by binding C3b and thus contributes to complement activation (Figures 3 and 4).

Our data underline the concept emerged from recent research that FHRs—in contrast to FH—rather promote complement activation (13, 14, 20–24). The FHRs may be involved in the fine-tuning of complement activation on ligands and surfaces exposed on altered self and during inflammation and complement attack, such as deposited C3b, the pentraxins PTX3 and CRP, ECM components, and dying cells. This capacity lies mainly in the conserved domains homologous to the main ligand binding sites for host and non-host molecules in FH (Figure 1A (8, 13)). Results obtained in the past years raise the possibility that the evolution of the *CFH* gene cluster and the appearance of the *CFHR* genes are microorganism-driven, due to coevolution with microbes, and the FHRs function as decoys to prevent FH binding by pathogens, thus blocking one of their escape mechanisms (42). At the same time, FHRs can compete with FH for self-ligands such as pentraxins, too [Figure 5A and (23, 24)]. In addition, similar to what was described for the well-known positive complement regulator properdin (43, 44), FHRs serve as a platform for alternative pathway convertase formation and are able to promote complement activation on host ligands and surfaces where they are bound (23, 24). Such an activity is shared by FHR-B; it can activate mouse complement and can also promote complement activation and C3 fragment deposition on mouse PTX3, CRP, and Matrigel (Figures 4 and 5).

Factor H contributes to the protection of various host cellular and non-cellular surfaces from the deleterious effects of excessive complement activation. The protective role of FH binding to endothelial cells and extracellular matrices is known from functional analyses of disease-associated FH mutations and FH autoantibodies, *in vitro* experiments and mouse models of diseases (12, 45–52). All these data point to a delicate balance of complement activation and inhibition necessary for homeostasis and prevention of harmful inflammation. The C-terminal domains of FH, especially the CCP20, are important for the interaction of the regulator with cell surface glycosaminoglycans and sialic acid as well as with C3b deposited during complement activation (30, 53–58). These domains are conserved among FHR proteins, including FHR-B. Previously, we showed binding of mouse FHRs from plasma to HUVEC surface (9). We extended those studies here and also investigated the complement activation on HUVEC-derived ECM, where FHR-B significantly increased the activation of complement and the deposition of C3 fragments (Figure 6). Similarly, FHR-B promoted complement activation on the murine ECM Matrigel (Figure 5B).

Mutations in FH or autoantibodies against its C-terminal domains cause reduced FH binding to host cells and this reduced protection results in complement-mediated damage. This can be measured *in vitro* by hemolysis assays using SRBCs (59, 60). We used this assay to compare the effect of exogenous mouse FH15–20 and FHR-B. Both molecules significantly enhanced SRBC lysis when added to mouse serum, but FHR-B was significantly more effective in that compared with FH15–20 (Figure 8). While both can bind to C3b and host cells and compete off FH, FHR-B bind to C3b stronger than does FH15–20 (Figure 2), and it also promotes complement activation directly (Figure 4), explaining its more prominent lytic effect.

Complement is integrally involved in the opsonophagocytic clearance of dead cells, and FH binding to the surface of apoptotic and necrotic cells contributes to the generation of proper amounts of opsonins without excessive complement activation, in order to avoid inflammation and lysis of the dead cells (61–63). FHR-5 and FHR-1 were recently shown to bind to necrotic cells and enhance complement activation, and thus opsonization of necrotic cells (24, 27). In the current study, we found that FHR-B, too, bound to necrotic cells and the bound FHR-B induced complement activation and deposition of C3 fragments on murine A20 B cell line and Jurkat T-cells when exposed to mouse serum (Figure 7).

A limitation of our study is the use of human C3b, ECM and necrotic cells as surrogate for their murine counterparts in some of the experiments. Because of the difficulty to have access to highly purified and well-characterized mouse C3b and other reagents, we opted to characterize FHR-B interactions with human C3b and HUVEC-ECM, while at the same time showing functional activity in the mouse system. We found this set-up also useful to demonstrate conservation of ligand binding and functional activities of FHRs (human C3b for binding and convertase assays—mouse C3 detection in functional assays; Matrigel as murine ECM model—HUVEC-ECM; necrotic Jurkat cells—necrotic murine A20 cells). This approach is supported and directly builds on previous reports describing and characterizing interactions of mouse FH, mouse FH CCPs 18–20 fragment and FHR-B with human C3b and HUVEC (9, 39).

In summary, our study is the first proving FH—FHR competition for a non-human FHR and the results support a general complement de-regulatory and FH-opposing role of the FHR proteins. We described new ligands of FHR-B, such as murine PTX3, CRP, ECM, and necrotic cells. Importantly, FHR-B induced complement activation itself and when bound on pentraxins, ECM and dead cells. These data support a conserved function among human and mouse FHR proteins and may guide study design and interpretation, as well as development of animal models for studying the *in vivo* function of FHR proteins.

## Author Contributions

MJ initiated and supervised the study. MC, ÁC, BU, and MJ designed the experiments. MH cloned, expressed, and purified recombinant proteins. MC, ÁC, ZM, and AK performed ligand binding and competition assays. ÁC performed convertase assays. MC, ÁC, and MH performed complement activation assays. MC and BU performed flow cytometry measurements. MC performed hemolysis assays. MC, BU, and MJ wrote the manuscript with the help of the other authors.

## Conflict of Interest Statement

The authors declare that the research was conducted in the absence of any commercial or financial relationships that could be construed as a potential conflict of interest.

## Abbreviations

BSA, bovine serum albumin; CCP, complement control protein; CRP, C-reactive protein; DPBS, Dulbecco’s phosphate-buffered saline; ECM, extracellular matrix; FHR, factor H-related; HUVEC, human umbilical vein endothelial cell; PTX3, pentraxin 3, FB, factor B; FH, factor H; FP, factor P; HSA, human serum albumin.
